# Awakening and Breathing Coordination, Delirium Monitoring and Early Mobility bundle in adult ICU patients: a preliminary cost analysis

**DOI:** 10.1186/cc13599

**Published:** 2014-03-17

**Authors:** G Peitz, K Dvoracek, J Sankaranarayanan, M Balas, K Olsen

**Affiliations:** 1 University of Nebraska Medical Center, Omaha, NE, USA; 2 Avera McKennan Hospital and University Health Center, Sioux Falls, SD, USA; 3 University of Conneticut/Hartford School of Pharmacy, Storrs, CT, USA; 4 The Ohio State University, Columbus, OH, USA

## Introduction

We previously demonstrated a significant increase in the number of ventilator-free days and reduced the rate of delirium in ICU patients treated with the Awakening and Breathing Coordination, Delirium Monitoring and Early Mobility (ABCDE) bundle. This study investigated whether implementation of the ABCDE bundle was cost- effective at an academic medical center.

## Methods

A *post-hoc *cost-effectiveness study was done following the before-after ABCDE bundle implementation study. The bundle consisted of the following components: daily spontaneous awakening trials (SATs); daily spontaneous breathing trials coordinated with the SATs; delirium monitoring; and early mobility. The study's primary endpoint was the cost-effectiveness of the ABCDE bundle in terms of the bundle's cost to prevent 1 day of mechanical ventilation (MV) or 1 day of delirium for all patients, as well as for a subgroup of non-MV patients. All cost-effectiveness ratios (CERs) were constructed from the hospital's cost perspective. The economic analyses were carried out in two steps. First, the mean cost per patient per hospital stay and total costs in the pre-ABCDE and post-ABCDE bundle periods were compared. Next, CERs of each respective primary endpoint were computed as incremental costs of implementing the bundle to prevent 1 day of MV and to prevent 1 day of delirium. *P *< 0.05 was considered statistically significant.

## Results

Data were analyzed from 146 and 150 patients in the pre- ABCDE and post-ABCDE groups respectively. There were mean decreases of 0.59 MV days and 0.83 delirium days between the pre and post bundle implementation. The mean costs (per patient per ICU stay) of implementing the bundle components were significantly higher in the post-ABCDE cohort versus the pre-ABCDE cohort ($191 vs. $92, *P *< 0.0001), with an additional increase in total costs ($8,930 vs. $8,624, *P *= 0.0233). Costs to prevent 1 day of MV and to prevent 1 day of delirium were $552 and $368 respectively. In the non-MV patients, the post-ABCDE bundle demonstrated fewer days of delirium than the pre- ABCDE cohort (0.8 vs. 1.13 days, *P <*0.05) but had higher overall costs ($5,417 vs. $4,546, *P *< 0.05), resulting in a cost of $2,639 to prevent 1 day of delirium in a non-MV patient.

## Conclusion

Implementing the ABCDE bundle in adult ICU patients appears to be a feasible cost-effective strategy when considering costs of mechanical ventilation and ICU delirium.

